# A biomolecular anthropological investigation of William Adams, the first SAMURAI from England

**DOI:** 10.1038/s41598-020-78723-2

**Published:** 2020-12-10

**Authors:** Fuzuki Mizuno, Koji Ishiya, Masami Matsushita, Takayuki Matsushita, Katherine Hampson, Michiko Hayashi, Fuyuki Tokanai, Kunihiko Kurosaki, Shintaroh Ueda

**Affiliations:** 1 Department of Legal Medicine, Toho University School of Medicine, 5-21-16, Omori-Nishi, Ota-ku, Tokyo, 143-8540 Japan; 2 Bioproduction Research Institute, National Institute of Advanced Industrial Science and Technology (AIST), Sapporo, 062-8517 Japan; 3 Computational Bio Big Data Open Innovation Lab (CBBD-OIL), National Institute of Advanced Industrial Science and Technology (AIST)-Waseda University, Tokyo, 169-8555 Japan; 4 The Organization of Anthropological Research, Yamaguchi, 759-6604 Japan; 5 Department of Biological Sciences, Graduate School of Science, The University of Tokyo, Tokyo, 113-0033 Japan; 6 Center for Accelerator Mass Spectrometry, Yamagata University, Kaminoyama, 999-3101 Japan

**Keywords:** Mitochondrial genome, Next-generation sequencing

## Abstract

William Adams (Miura Anjin) was an English navigator who sailed with a Dutch trading fleet to the far East and landed in Japan in 1600. He became a vassal under the Shogun, Tokugawa Ieyasu, was bestowed with a title, lands and swords, and became the first SAMURAI from England. "Miura" comes from the name of the territory given to him and "Anjin" means "pilot". He lived out the rest of his life in Japan and died in Hirado, Nagasaki Prefecture, in 1620, where he was reportedly laid to rest. Shortly after his death, graveyards designated for foreigners were destroyed during a period of Christian repression, but Miura Anjin’s bones were supposedly taken, protected, and reburied. Archaeological investigations in 1931 uncovered human skeletal remains and it was proposed that they were those of Miura Anjin. However, this could not be confirmed from the evidence at the time and the remains were reburied. In 2017, excavations found skeletal remains matching the description of those reinterred in 1931. We analyzed these remains from various aspects, including genetic background, dietary habits, and burial style, utilizing modern scientific techniques to investigate whether they do indeed belong to the first English SAMURAI.

## Introduction

**Figure 1:**
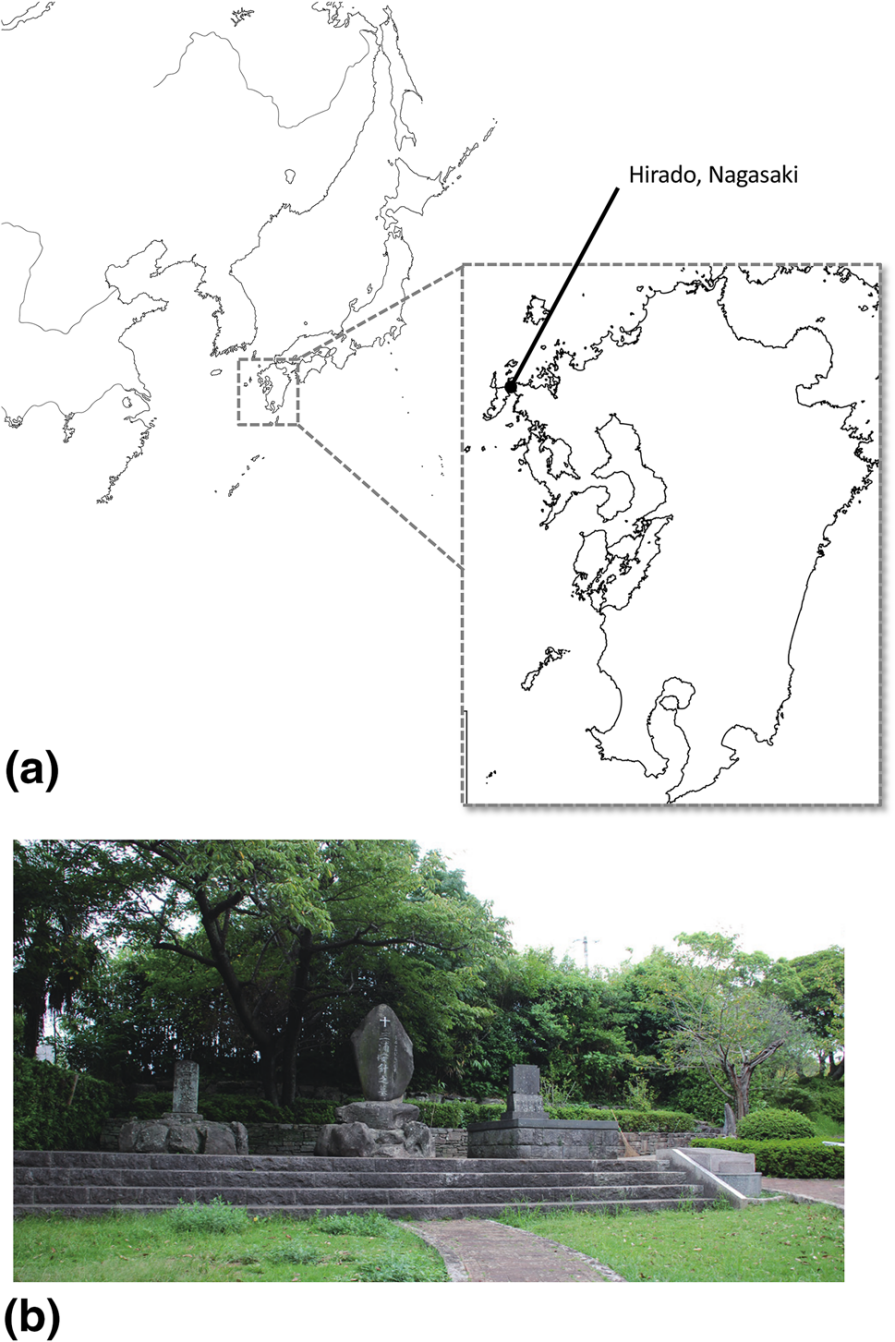
(**a**) A map showing the location of Hirado City, Nagasaki Prefecture, Japan, where Miura Anjin’s grave is situated. Base map sourced from https://www.freemap.jp. (**b**) An image of the tombstone at Miura Anjin’s grave.

William Adams was born in Gillingham, England in 1564. He worked in a shipyard from the age of 12, and later studied shipbuilding techniques, astronomy and navigation. He also experienced Arctic expeditions and naval captaincy. At the age of 34, he embarked on an expedition to the orient by a private Dutch fleet as chief officer. The voyage encountered numerous disasters, and of the five ships that set sail, only the one in which he eventually sailed on, the Liefde, remained. With a few survivors onboard the Liefde landed in present-day Oita Prefecture in Japan in 1600. The Shogun, Tokugawa Ieyasu seized power and established a government at Edo (now known as Tokyo) in 1603. Ieyasu took on Adams as an advisor and studied mathematics and geography from him. He also heavily relied on Adams as a diplomatic advisor to the Shogunate. Adams was requested by Ieyasu to build a Western-style sailing ship, and in 1604 he built the first shipbuilding dock in Japan in Ito. In 1607, in response to Adams's achievements, Ieyasu selected him for the high-prestige position as a direct retainer in the Shogun's court, entrusting him with Miura-gun (now a part of Yokosuka City) territories and swords. He was bestowed the name Miura Anjin. "Miura" comes from the name of the territory, and "Anjin" means "pilot". This made him the first Englishman to hold the position of SAMURAI. He had the unusual situation of living as a Japanese SAMURAI despite being a foreigner. He worked for the Shogunate's overseas trade industry, including the establishment of the Hirado Trading House in the Netherlands and the United Kingdom (1613). When the British East India Company ship Clove arrived in Hirado in 1614 Adams had a chance to return to England, but he opted to stay in Japan to live out the rest of his days. Adams reportedly died in 1620 in Hirado, Nagasaki Prefecture (Fig. 1a), and was buried in a cemetery designated for foreigners in 1620, due to the changing times in Japan in the Early Edo period (1600–1868). After the Shimabara rebellion in 1637, shortly after Adams’ death, foreign graveyards were destroyed during a period of Christian repression. Legend says that his bones were, however, saved by a close family friend and reburied. In 1931, a grave marked as a Miura family tomb was excavated^1^. During the excavation, fragments of crania, scapulae, vertebrae, ribs, femurs, and one molar were unearthed along with one piece of earthenware, and 30 corroded nails. The bones were found directly under the gravestone in order; crania–upper body–upper limbs–spine–pelvis–lower limbs, and it was estimated that they were interred in a coffin (around 190 cm long and 65 cm wide). From the position of the head and feet, it was recorded that the individual would have been around 170 cm tall^1^. After the excavation, the bones were reburied at the current site, where a tombstone dedicated to Miura Anjin (William Adams) now stands (Fig. 1b). During archaeological excavations in 2017, human skeletal remains matching the description of those found in 1931 were uncovered.

## Results and discussion

Approval for the present study was provided by the Ethics Committee of Toho University School of Medicine (A18101_A18058) and relevant guidelines were followed for the study.

### Site and excavation

Archaeological excavations were conducted in Hirado City, Nagasaki Prefecture, Kyushu, Japan in 2017 (Fig. 1a). During excavations directly under the tombstone (Fig. 1b), a porcelain vase was excavated from under a layer of gravel, and skeletal remains belonging to a single individual were found inside of it. The skeletal remains consisted of the skull, mandible, femur, and part of the tibia, and match the description of those from the 1931 report. Inspection of the layer under the burial revealed markings of a rectangular grave (Fig. S1ab).

**Figure 2:**
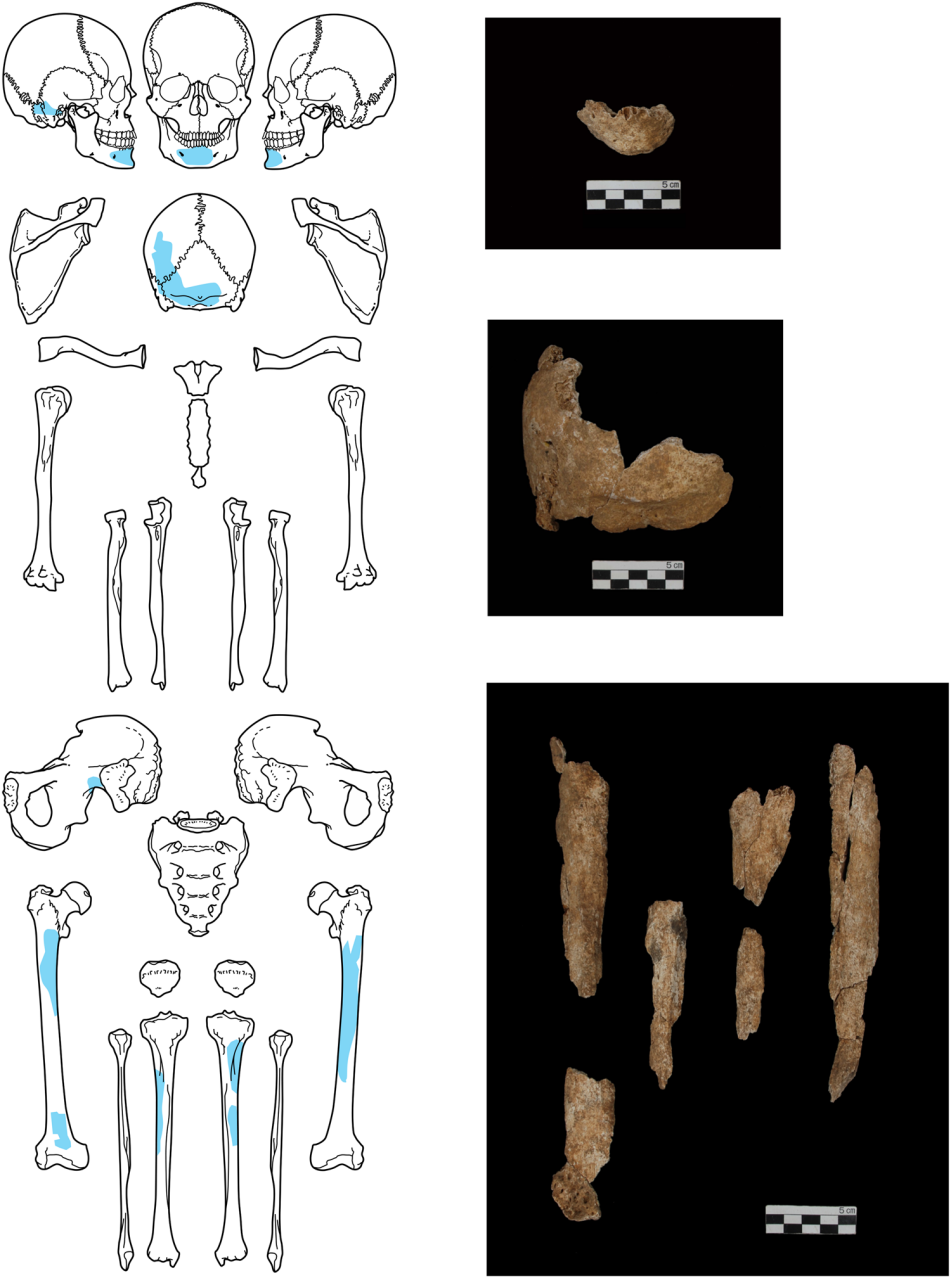
The fragments of human bone recovered during the excavation are colored blue. The diagram shows the mandible at the top, the skull in the middle, and the long bones below.

The remains consist of a part of the cranium, mandible, femur, tibia, and bone fragments (Fig. 2). There were no accompanying grave goods indicating the identity of the remains. However, the grave pit detected in 1931 was a rectangular cuboid shape, and so it is probable that a coffin was used, and the burial posture was extended supine. In the Edo period, circular shape graves were the norm, and extended supine burials were only used for Westerners or Christians, not for most native Japanese. The grave matches the description of a burial pit with the remains of skeletal remains and a coffin found and reinterred in 1931^1^. In addition, the grave site has been protected as one of a nobleman by the people living in Hirado for centuries.

### Radiocarbon dating and stable isotopic measurements

The stable carbon isotope ratio was − 18.87‰ and the stable nitrogen isotope ratio was + 11.95‰ for the collagen sample. The carbon content of the collagen was 15.82%, the nitrogen content was 4.85%, and the atomic carbon/nitrogen ratio was 3.8. This value was different from the recommended one (2.9–3.6) obtained for well-preserved bone collagen^2^. This result indicates that the sample was affected by contamination of biotic substances such as humic acids.

The radiocarbon age was dated as 410 ± 30 years BP (Beta-495523, Conventional ^14^C age). Since humans intake some of their proteins from marine products, bone collagen contains some marine carbon. Hence, it is necessary to consider a marine reservoir effect to obtain a calendar age from bone collagen. The ratio of marine resources was set to 25.1% from the carbon isotope ratio (δ^13^C) following the methodology of Arneborg et al.^3^. The ratio is applied to obtain the modelled calibration curve, which comprises a combination of the international calibration datasets of IntCal20^4^ and Marine20^5^. Then, the calendar age is calculated using OxCal 4.4^6^. The influence of the local marine reservoir was taken from the data for nearby Hakata Bay, HKA2-1 1.0–1.2 m^7^. The local marine reservoir effect is ΔR = 53 ± 63 (^14^C year) which is derived by using the 14 CHRONO Marine Reservoir Database (http://calib.org/deltar/). The obtained calendar dates (calibrated ages with 95.4% probability) are calAD 1466–1819 (92.0%), 1836–1867 (1.6%), and 1925–1948 (2.0%) (Fig. S2). Although the atomic C/N ratio is outside the recommended values, the calibrated calendar age is consistent with "Adams died in Hirado, on 16 May 1620, at the age of 55".

The ratios of stable carbon and nitrogen isotopes of collagen extracted from human skeletal remains reflect the isotopic ratios of the food ingested by the individual, and thus can be used to estimate dietary habits (e.g., dependence on marine products, C3 plants, C4 plants, etc.). Figure S3 shows plots of the carbon and nitrogen stable isotope ratios on an isotope distribution map. The stable isotope ratios of carbon and nitrogen for human skeletal remains from the Edo period were obtained using the remains of 149 individuals from Edo^8,9^ and 21 individuals from Kyoto^10^. The value for the specimen was included among the Japanese cluster for the Edo period. This means that the individual consumed the same diet as the Japanese in the Edo period.

### Ancient DNA analysis

**Figure 3:**
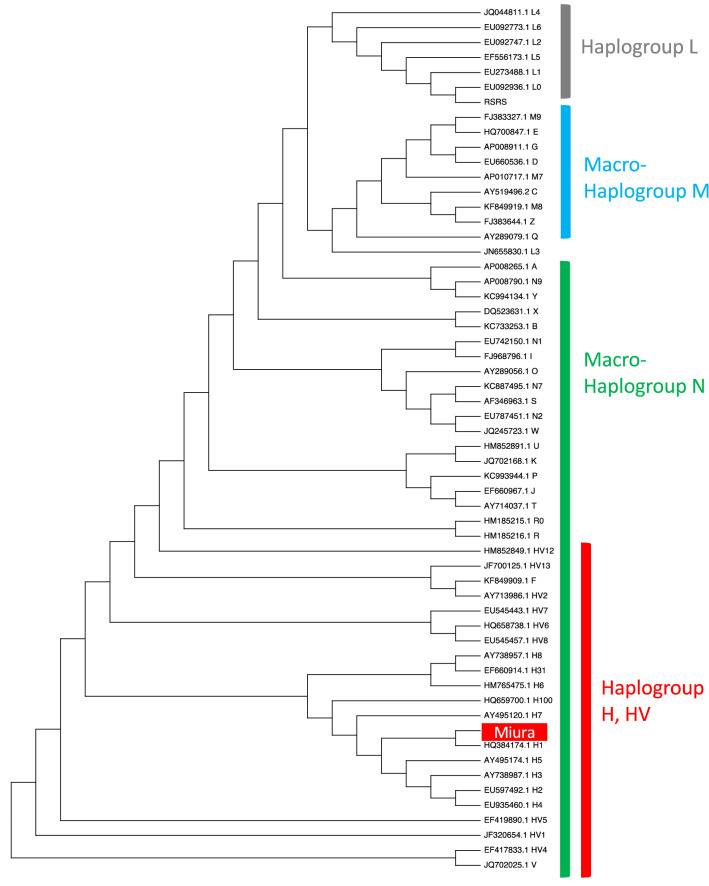
A Maximum-Parsimony tree for mtDNA sequences of Miura Anjin and 57 worldwide mtDNA sequences belonging to haplogroups L, M, A, B, C, D, E, F, G, H, I, J, L, M, N, O, P, Q, R, S, T, V, W, X, Y, and Z. The accession number and haplogroup are described in the phylogenetic tree.

Due to poor DNA preservation, it was very difficult to obtain sufficient DNA sequences for Single Nucleotide Polymorphism (SNP) analysis such as DNA phenotyping and Y-haplotyping, although whole genome enrichment. However, we obtained nucleotide sequences of mitochondrial DNA (mtDNA) for the individual with an average depth of coverage of 10x. The percentage of mtDNA coverage was 96.4%. We observed the diagnostic feature of ancient DNA for this human remains, a pattern of cytosine to thymine substitutions (deamination) at the 5′ and 3′ end of the sequence reads, which provides the authenticity of DNA sequences obtained (Fig. S4). We observed all of the sites that designate haplogroup H1e2b. We also obtained a phylogenetic tree by combining the mtDNA sequence of this individual with those of 57 worldwide haplogroups in the world. It was confirmed that the sequence was most closely related to those belonging to haplogroup H (Fig. 3). This haplogroup H has been observed very frequently in European populations, and, now accounts for over 40% of mtDNA variations in modern humans across much of Western Eurasia, with declining frequencies south and east to ~ 10–30% in the Near East and Caucasus^11,12^. In particular it is characteristic of Western and Northern European populations. Haplogroup H has not been observed in any Japanese populations. It has also not been observed in Korean or Chinese East Asian populations. Based on the above findings, the skeletal remains can be considered to have originated from any population in Western and Northern Europe, including the United Kingdom.

As a whole, our investigations disclosed the bioarchaeological characteristics of the grave and the skeletal remains discovered within, and it can be strongly considered that they are those of a Westerner, likely Miura Anjin. The mtDNA analysis indicates haplogroup H that has been observed very frequently in characteristic of Western and Northern European populations but not among Japanese. The result of the radiocarbon dating is consistent with the historical record stating "Adams died in Hirado, North of Nagasaki, on 16 May 1620, at the age of 55". Finally, stable isotope analyses show that despite being of European decent, the person had lived in Japan for a long time.

Although the evidence is not unequivocal, the combination of the results obtained does not disprove the notion that the skeletal remains belong to Miura Anjin. The only caveat is that if the original burial was in a graveyard for foreigners, there may be a chance that the bones are from another individual buried there with similar characteristics to Miura Anjin. In order to fully confirm the identity of the remains, a future study should incorporate a comparison of autosomal DNA with a living relative of Miura Anjin (if a relative can be found), like that performed during investigations of Richard III of England’s remains^13^.

## Methods

### Radiocarbon dating and stable isotopic measurements

After the femoral fragments were alkali-washed, collagen was extracted. Radiocarbon dating and stable isotopic measurements were conducted at the Beta Analytic Radiocarbon Dating Laboratory, Miami, Florida (Beta-495523). ^14^C dates were converted into calibrated age by comparison with the international calibration datasets of IntCal20^4^ and Marine20^5^. Stable isotope ratios of carbon and nitrogen were measured using an isotope ratio mass spectrometer (IRMS) combined with elemental analysis (EA) to produce N_2_ and CO_2_ sequentially. The ^13^C/^12^C and ^15^N/^14^N ratios were shown as delta values (δ^13^C and δ^15^N) in comparison with international standards.

### DNA extraction, NGS library preparation, enrichment and sequencing

DNA was extracted from bone, and next generation sequencing (NGS) library was constructed according to our previous methods^14^ with slight modifications as follows^15^.

#### DNA extraction

DNA was extracted from a part of the right petrous bone (Fig. S5). Extraction was performed using recognized standards followed by the authors in previous studies^14,15^. After exhaustively brushing to eliminate dirt and exogenous contaminants, the outer bone surface was mechanically removed with a sanding machine (Dremel) to further remove surface contaminants. The clean bone was cut into small pieces of approximately 1–2 cm^3^ using an electric drill cutter (Dremel). The bone fragments were cooled with liquid nitrogen, and fine powdered bone was obtained by grinding bone fragments in a mill (Multi-beads Shocker MB601U, YASUI KIKAI).

During all steps of DNA extraction, NGS library preparation, and enrichment, we took all possible precautions to guard against contamination. Experiments were performed in a laboratory that is exclusively dedicated to ancient DNA work and is physically isolated from other molecular work laboratories. All manipulations were performed in a laminar flow cabinet routinely irradiated with UV light. Frequent surface cleaning was routinely performed before and after working. A facemask, head cap, and clean laboratory coat were always worn, and gloves were frequently replaced. All the procedures were conducted using new sterilized disposable tubes and filter pipette tips. All non-disposable glass and metallic materials were dry-heat sterilized at 160 °C for 2–4 h.

#### NGS library preparation

We performed DNA preparation and NGS library construction according to our previous methods^14^ with slight modifications as follows. The powdered samples were digested in 0.5 M EDTA (pH 8.0) for 2 h at 56 °C in a rotating hybridization oven, and the supernatant was removed by centrifugation. This decalcification step was repeated three times in total. DNA was extracted with phenol:chloroform:isoamyl alcohol (25:24:1), followed by extraction with an equal volume of chloroform. After centrifugation, the aqueous solution was removed and subsequently concentrated by centrifugation dialysis using an Amicon Ultra-15 30 kDa centrifugal filter (Merck Millipore) to a final volume of 200 μL. The DNA solution was purified with silica-based MiniElute spin columns (Qiagen) according to manufacturer's protocol. The obtained DNA was quantified using Quant-iT dsDNA HS assay kit (Thermo Fisher Scientific). Using the obtained DNA, we prepared single- and double-stranded libraries.

#### Enrichment and sequencing

Libraries were enriched for whole human genomes, using in-solution target enrichment (myBaits, Arbor Biosciences), or for human mitochondrial DNA (mtDNA) using in-solution target enrichment (SureSelect, Agilent Technologies). These enriched libraries were pooled sequenced on an Illumina MiSeq.

### NGS data processing and ancient DNA authentication

Raw sequencing reads were trimmed by removing adapter sequences and low-complexity sequences with fastp ver. 0.20.0^16^. The trimmed reads were mapped against the human mtDNA reference sequence (rCRS)^17^ using the Burrows-Wheeler Alignment tool (BWA)^18^ with optimal parameters for ancient DNA^19^. Reads with a mapping quality lower than 30 were filtered out and high-quality mapped reads were retained using SAMtools ver. 1.9^20^. To minimize the effects of nucleotide mis-incorporations on building a consensus mtDNA sequence, the first two bases on each end of the read were clipped with BamUtil ver. 1.0.14^21^ according to our previous bioinformatics procedure^22^. Then, reads with a significant hit (E-value <  = 1e − 15) to non-human genomes (e.g., fungal or bacterial genomes) were identified by BLASTN and then were also filtered out. In addition, reads mapped to human nuclear genome sequences of hg19 were also removed as numts (nuclear copies of mtDNA). Mismatch percentage was estimated using MitoSuite ver.1.0.9^23^. Finally, consensus sequences were built using MitoSuite, and their mtDNA haplogroup assignments were called with HaploGrep2 (https://haplogrep.uibk.ac.at/)^24^. We checked variants using IGV software^25^ based on PhyloTree Build 17 (http://www.phylotree.org/)^26^.

### Sequence and phylogenetics

The nucleotide sequences were aligned to rCRS using MAFFT^27^ and manually checked. Owing to hypervariability, the A/C stretch length polymorphisms at nucleotide positions (nps) 303–315 and 522–523, and variation at np 16519 were disregarded for tree reconstruction. The A/C stretch length polymorphism at nps 16180–16193 was also disregarded, except for variation at np 16189, which is one of the defining variations of haplogroup B. A Maximum-Parsimony tree was obtained by MEGA program, version X^28^.

## Supplementary Information

Supplementary Figures.

## Data Availability

The nucleotide sequences obtained were deposited in GenBank under accession numbers LC592620, LC592621.
