# Supplementary material for: A biomolecular anthropological investigation of William Adams, the first SAMURAI from England
Source: Sci Rep. 2020 Dec 10;10:21651. doi: 10.1038/s41598-020-78723-2 (PMC7729870; doi:10.1038/s41598-020-78723-2)
Supplement: Supplementary file 1 — Supplementary Figures. [file 41598_2020_78723_MOESM1_ESM.pdf]

A biomolecular anthropological investigation of William Adams, the first SAMURAI from England

Supplementary Information

Fuzuki Mizuno[1*], Koji Ishiya[2, 3], Masami Matsushita[4], Takayuki Matsushita[4], Katherine Hampson[5], Michiko Hayashi[1], Fuyuki Tokanai[6], Kunihiko Kurosaki[1*], and Shintaroh Ueda[1,5]

[1] Department of Legal Medicine, Toho University School of Medicine, Tokyo 143-8540, Japan

[2] Bioproduction Research Institute, National Institute of Advanced Industrial Science and Technology (AIST), Sapporo, 062-8517, Japan

[3] Computational Bio Big Data Open Innovation Lab (CBBD-OIL), National Institute of Advanced Industrial Science and Technology (AIST)—Waseda University, Tokyo, 169-8555, Japan

[4] The Organization of Anthropological Research, Yamaguchi 759-6604, Japan

[5] Department of Biological Sciences, Graduate School of Science, The University of Tokyo, Tokyo 113-0033, Japan

[6] Center for Accelerator Mass Spectrometry, Yamagata University, Kaminoyama, 999-3101, Japan

*Correspondence1 to Fuzuki Mizuno
Address: 5-21-16, Omori-Nishi, Ota-ku, Tokyo 143-8540, Japan
Tel: +81-3-3762-4151, E-mail: fuzuki.mizuno@med.toho-u.ac.jp

*Correspondence2 to Kunihiko Kurosaki
Address: 5-21-16, Omori-Nishi, Ota-ku, Tokyo 143-8540, Japan
Tel: +81-3-3762-4151, E-mail: kurosaki@med.toho-u.ac.jp

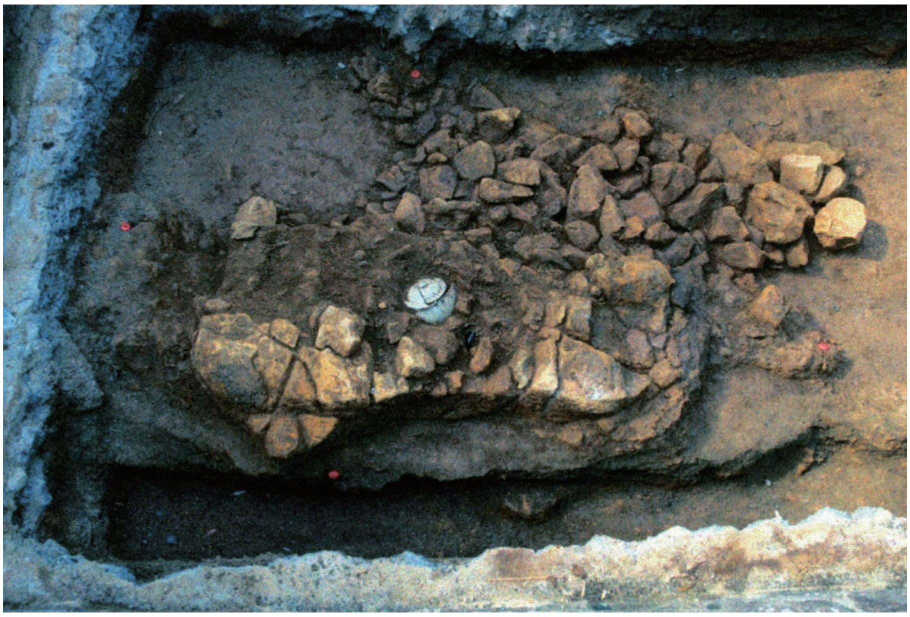

Figure S1 (a)

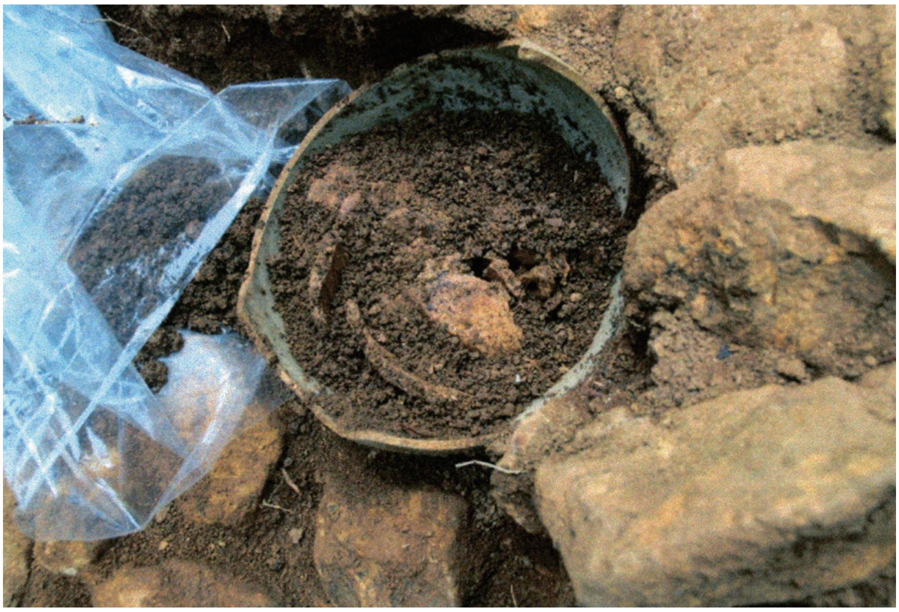

Figure S1 (b)

Figure S1

(a) The excavation under the gravestone of Miura Anjin.

(b) A porcelain vase excavated from the tomb. Human skeletal remains were found inside the vase, likely those reinterred in 1931.

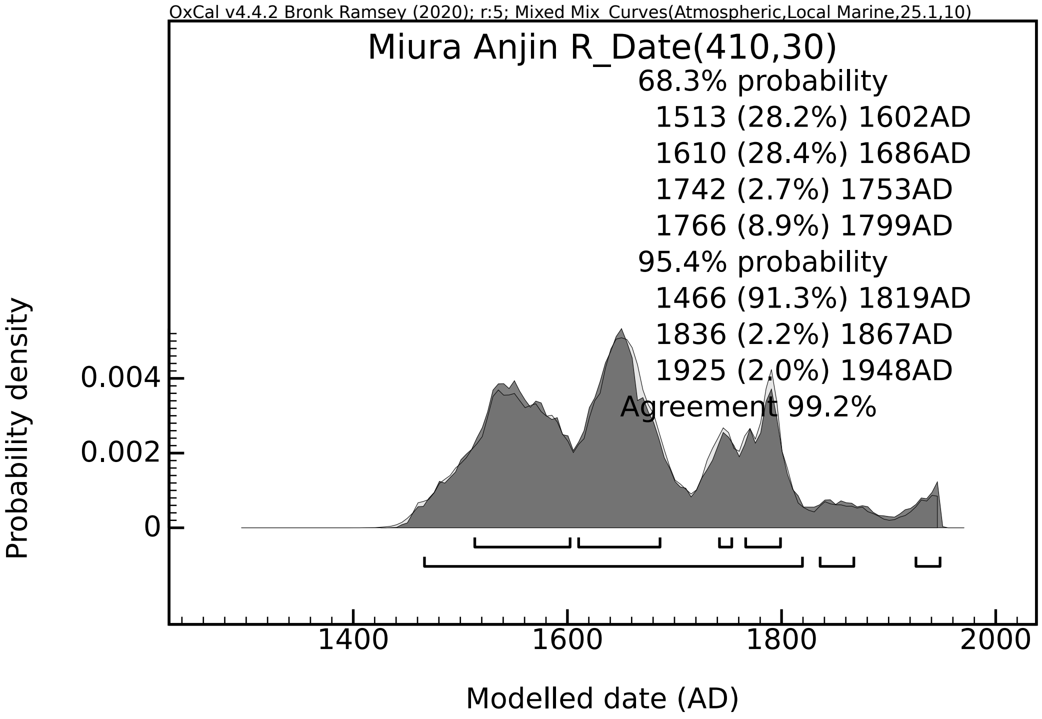

Figure S2

Modelled date of Miura Anjin, after applying the marine reservoir correction and calibration in OxCal by mixing the IntCal20 and Marine20 calibration curves.

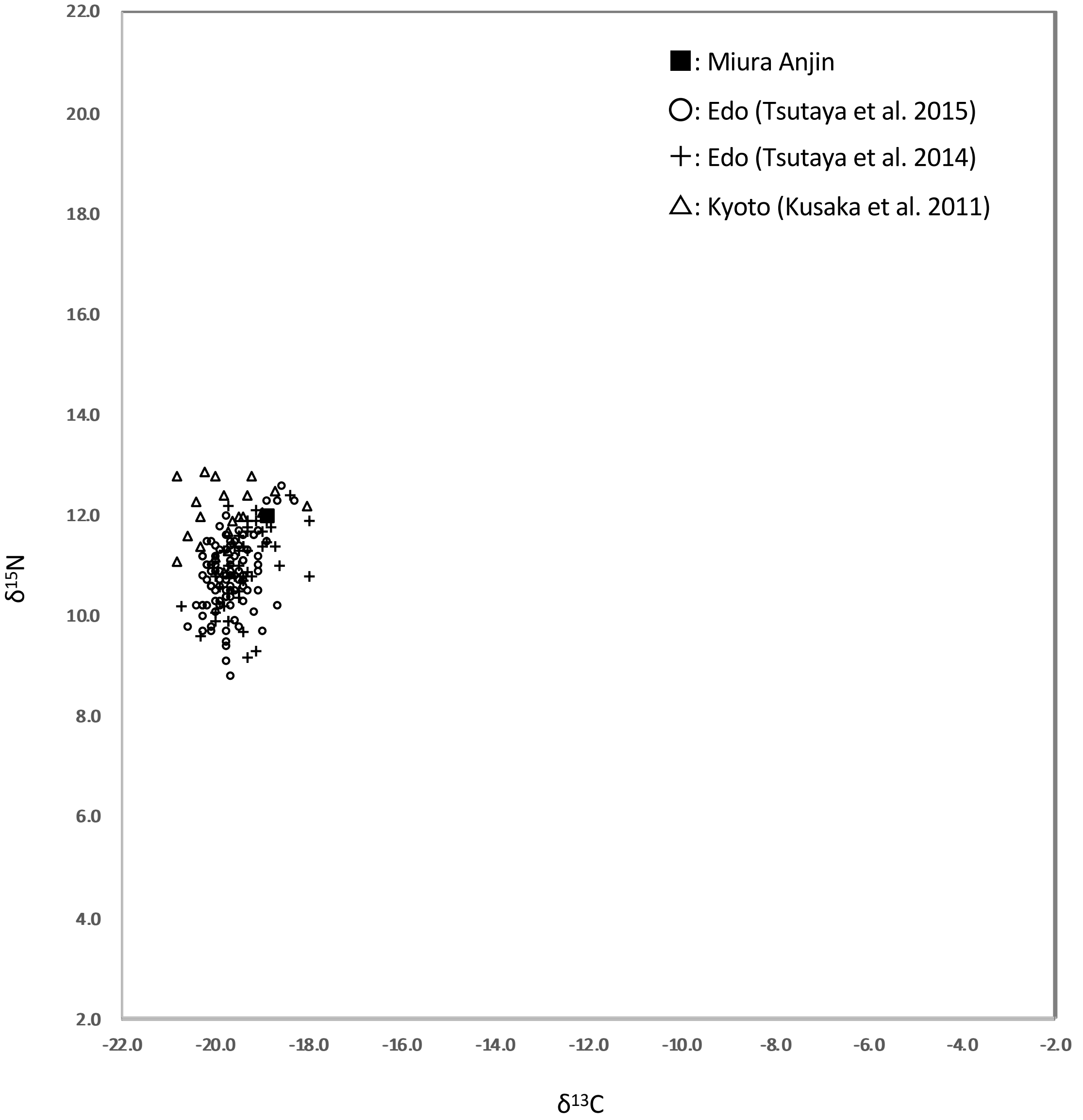

Figure S3

A plot of the carbon and nitrogen stable isotope ratios on an isotope distribution map.

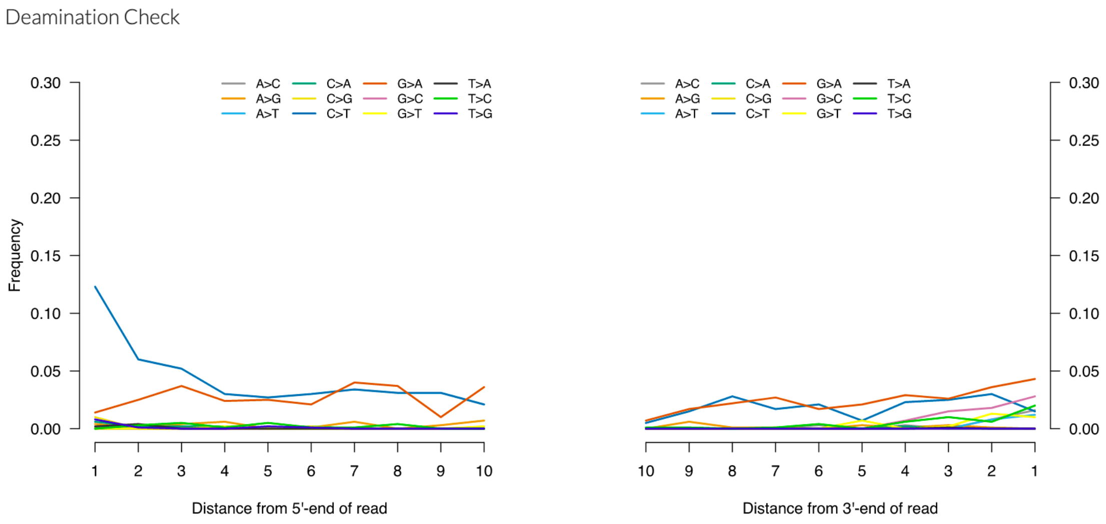

Figure S4

Misincorporation plot obtained using MitoSuite.

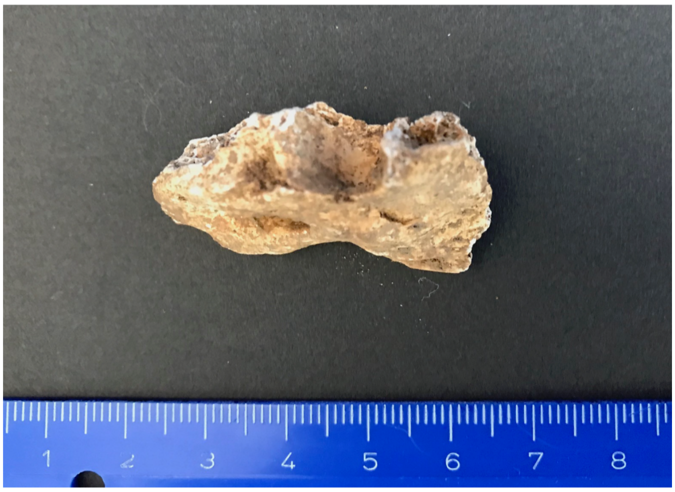

Figure S5

The right petrous bone used for DNA analysis.
